# A comprehensive review on optical and electrochemical aptasensor for detection of fumonisin B1

**DOI:** 10.3389/fnut.2025.1596673

**Published:** 2025-07-31

**Authors:** Jiayu Ma, Xiaodong Guo

**Affiliations:** ^1^College of Animal Science and Technology, Yangzhou University, Yangzhou, China; ^2^Joint International Research Laboratory of Agriculture and Agri-Product Safety of MOE, Yangzhou University, Yangzhou, China

**Keywords:** fumonisin B1, aptasensors, optical signal, electrochemical signal, food safety

## Abstract

Fumonisin B1 (FB1) contamination has emerged as a global concern due to its high incidence, severe toxicity, and profound implications for food safety and human health. Consequently, there is an urgent demand for the development of novel analytical techniques that enable simple, rapid, and accurate detection of FB1. Conventional methods for mycotoxin analysis, such as high-performance liquid chromatography (HPLC), liquid chromatography-mass spectrometry (LC–MS), thin-layer chromatography (TLC), gas chromatography–mass spectrometry (GC–MS), and enzyme-linked immunosorbent assay (ELISA), often suffer from limitations including high cost, time-consuming procedures, environmental sensitivity, and reliance on specialized expertise. Nucleic acid aptamers, generated via Systematic Evolution of Ligands by Exponential Enrichment (SELEX), have garnered significant attention as next-generation bioreceptors, demonstrating remarkable progress in food safety applications. Leveraging their high specificity and strong affinity for target molecules, aptamers have been successfully employed as alternatives to conventional methods for FB1 detection, leading to the development of diverse aptasensor platforms. This review systematically summarizes recent advancements (2013–2025) in optical and electrochemical aptasensors for FB1 detection, elucidating their working principles, merits, and limitations. It further evaluates the impact of material integration on sensor performance, identifies existing limitations in selected aptasensor configurations, and proposes corresponding optimization strategies. Finally, the current challenges hindering the practical implementation of aptasensors are critically analyzed, and future research directions are outlined to advance this promising field.

## 1 Introduction

The contamination of food and feed by mycotoxins represents a significant global challenge (1, 2). Mycotoxins, a group of highly toxic secondary metabolites produced by filamentous fungi (3, 4), exhibit potent carcinogenic, immunotoxic, and cytotoxic effects, posing severe risks to human health (5). Common mycotoxins, including aflatoxin B1 (AFB1), ochratoxin A (OTA), fumonisin B1 (FB1), and zearalenone (ZEN), are frequently detected in cereals and cereal-derived products (6, 7). Among fumonisins, FB1, the most prevalent and toxic variant, is a water-soluble toxin capable of inducing oxidative stress in cells and has been classified as a Group 2B carcinogen by the International Agency for Research on Cancer (IARC) (8, 9). Consequently, the development of simple, rapid, and accurate biosensors for FB1 detection is of critical importance.

Biosensor typically comprises five key components: the target analyte, biorecognition element (BRE), signal transduction element (STE), electronic system, and display interface (10). The BRE and STE are fundamental functional units. The BRE consists of two affinity partners, such as antigen/antibody or aptamer/target pairs. Common BREs include enzymes (11), antibodies (12), nucleic acids (13), cells (14) and aptamers (Apts) (15), all of which exhibit high specificity and selectivity toward their respective targets. The STE detects interactions between affinity pairs and converts biological signals into physically measurable outputs, such as optical (16), resistive (17), electrochemical (18), or photoelectrochemical signals (19).

Conventional mycotoxin detection methods are broadly categorized into chromatographic and immunological techniques. Chromatographic approaches, including high-performance liquid chromatography (HPLC), liquid chromatography-mass spectrometry (LC–MS), thin-layer chromatography (TLC), and gas chromatography–mass spectrometry (GC–MS), rely on instrumental platforms (20, 21). However, these methods demand specialized personnel, isolated laboratory facilities, costly reagents, and time-intensive sample preparation. In contrast, immunoassays, such as enzyme-linked immunosorbent assay (ELISA), immunosensors, and immunochromatography, offer simplicity and rapidity. While ELISA is widely adopted, it suffers from limited automation, prolonged detection times, and moderate sensitivity. Furthermore, ELISA depends on antigen–antibody interactions, where antibodies, as functional proteins, are environmentally sensitive, prone to denaturation, and exhibit high immunogenicity. Their limitation in recognizing small molecules, highly toxic targets, or low-immunogenicity compounds further restricts broader applications (22).

Apts, single-stranded DNA (ssDNA) or RNA molecules with specific molecular recognition capabilities, are screened via the Systematic Evolution of Ligands by Exponential Enrichment (SELEX) technique (23). Under specific conditions, Apts fold into unique three-dimensional structures, incorporating hairpins, G-quadruplexes, bulges, or pseudoknots, to bind targets with high specificity (24, 25). Compared to traditional antibodies, Apts offer advantages such as extended shelf life, minimal batch-to-batch variability, low/no immunogenicity, and flexibility in chemical modifications to enhance stability and affinity (26). These attributes have driven the widespread development of aptasensors for detecting diverse targets, including proteins, cells, viruses, bacteria, metal ions, and small-molecule mycotoxins. Among aptasensors, optical and electrochemical transducers have emerged as preferred platforms due to their versatility (10).

**Figure 1:**
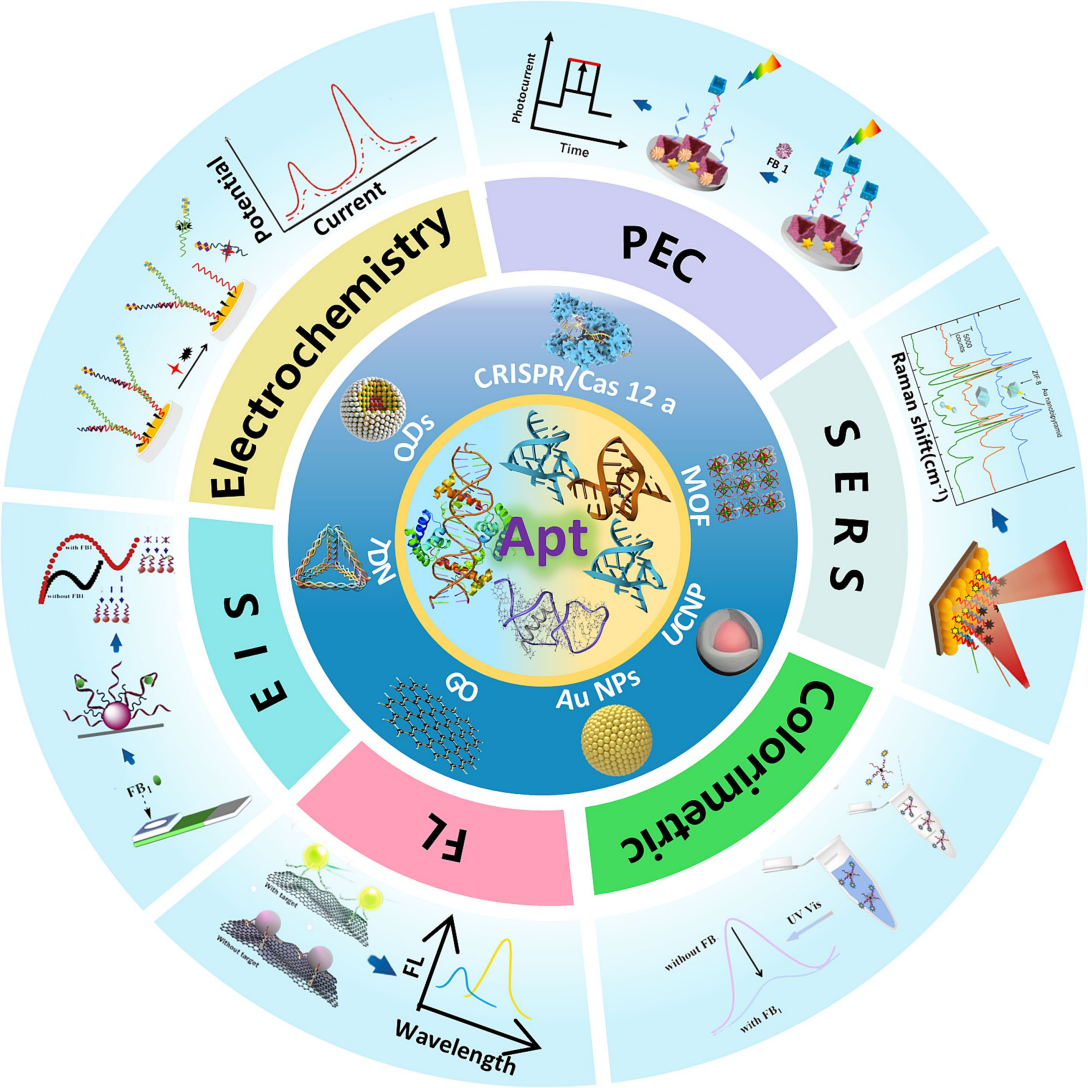
Comprehensive overview of aptamer based optical and electrochemical sensing of FB1.

Recent progress in mycotoxin detection research has been extensively documented. Comprehensive reviews addressing optical and electrochemical aptasensors for AFB1 (15, 27), OTA (28), and ZEN (29), as well as broader discussions on multi-mycotoxin detection strategies (30–32), are available. While liquid chromatography (33, 34), indirect competitive enzyme-linked immunosorbent assay (IC-ELISA) (35), and molecularly imprinted polymer solid-phase extraction (MIPSPE) (36) for FB1 analysis have been thoroughly explored, aptamer-based FB1 detection platforms remain insufficiently reviewed. Although Mirón-Mérida et al. (37) summarized aptasensors for FB1 in 2021, the rapid advancements in this field necessitate an updated and comprehensive review. This article systematically examines the progress in optical and electrochemical FB1 aptasensors from 2013 to 2025, detailing their working principles, strengths, and limitations. It further evaluates the impact of material integration on sensor performance, proposes solutions to existing challenges in aptasensor design, and concludes with an analysis of current obstacles and future directions for advancing aptasensor technology (Figure 1).

## 2 Applications of aptasensors for FB1 detection

### 2.1 Optical aptasensors for FB1 detection

Optical biosensors detect targets by recognizing changes in optical properties and converting them into readable signals (38). Owing to their rapid response, simplicity, high sensitivity, and cost-effectiveness, optical aptasensors have attracted extensive research attention and are widely applied in toxin detection. These sensors employ aptamers as biorecognition elements and utilize diverse optical transduction methods, including fluorescence (FL) (39), colorimetry (40), Surface plasmon resonance (SPR) (41), and surface-enhanced Raman spectroscopy (SERS) (42) (Table 1).

**Table 1:** Optical and electrochemical aptasensors for detection of FB1.

Aptasensor	Samples	FB1/signal	LOD	Dynamic range	References
FL	Wheat	Direct ratio	0.15 ng/mL	0.5–20 ng/mL	(39)
	Peanut	Direct ratio	16.2 pg/mL	50 pg/mL – 300 ng/mL	(45)
	Maize	Inverse ratio	16.84 nM	25–500 nM	(47)
	Cornstarch	Inverse ratio	0.802 ng/mL	10–1500 ng/mL	(48)
	Corn flour, oat flour and wheat flour	Direct ratio	0.45 pg/mL	1 pg/mL – 100 ng/mL	(49)
	Maize	Direct ratio	0.003 ng/L	0.01–100 ng/L	(53)
	Corn flour, oatmeal and infant supplements	Direct ratio	0.0121 ng/mL	0.032–500 ng/mL	(57)
Colorimetric	Wheat and corn	Direct ratio	0.38 pg/mL	5 × 10^−4^ – 50 ng/mL	(40)
	Maize and wheat	Direct ratio	0.024 ng/mL	0.05–100 ng/mL	(64)
	Maize	Inverse ratio	2.7 pg/mL	0.01–2000 ng/mL	(65)
	Beer and maize	Inverse ratio	0.3 ng/mL	0.5–300 ng/mL	(68)
	Maize	Direct ratio	–	10^−3^ – 10 ng/mL	(69)
SERS	Maize, onions, wheat and milk	Inverse ratio	0.05 ng/mL	0.1–1000 ng/mL	(73)
	Maize	Inverse ratio	3 pg/mL	10–500 pg/mL	(42)
EC	Rice	–	0.306 fg/mL	0.500 fg/mL – 1 ng/mL	(75)
	–	Direct ratio	–	0.5–500 pg/mL	(76)
	Beer	Inverse ratio	0.26 pg/mL	1.0 pg/mL – 100 ng/mL	(77)
	Maize	Direct ratio	20 pg/mL	50 pg/mL – 50 ng/mL	(82)
	Beer and maize	Inverse ratio	0.15 pg/mL	1.0 × 10^−3^ – 1000 ng/mL	(79)
	Maize	Direct ratio	5 × 10^−4^ ng/mL	1 × 10^−3^ – 1 × 10^−2^ ng/mL	(80)
PEC	Skim milk	Inverse ratio	0.016 pg/mL	0.001–100 ng/mL	(19)
	Corn paste	Direct ratio	0.13 pg/mL	1.0 × 10^−3^ – 1.0 × 10^2^ ng/mL	(87)
	Corn juice and vinegar	Direct ratio	0.10 pg/mL	1 pg/mL – 100 μg/mL	(88)
	Vinegar and sauces	–	2.7 pg/mL	10 pg/mL – 1000 ng/mL	(89)
	Beer and maize	Direct ratio	0.0723 pg/mL	1 × 10^−4^ – 1 × 10^2^ ng/mL	(90)
	Maize and flour	Direct ratio	65 fg/mL	0.1 pg/mL – 10 ng/mL	(91)
	Maize and soybean	Direct ratio	4.9 fg/mL	100 fg/mL – 1 μg/mL	(92)
EIS	Maize	Direct ratio	2 pM	0.1 nM – 100 μM	(93)
	Maize	Direct ratio	3.4 pg/mL	10 pg/mL – 50 ng/mL	(97)
	Cornmeal	Direct ratio	2.47 ng/mL	5–1000 ng/mL	(17)
ECL	Maize and peanut	Inverse ratio	–	5 × 10^−5^ – 0.5 ng/mL	(101)

#### 2.1.1 Fluorescent aptasensor

Fluorescent aptasensors are among the most common methods for detecting mycotoxins in food. Simple fluorophore/quencher pairs (e.g., FAM/Dabcyl) are often used in fluorescence quenching assays. Since naturally fluorescent biomolecules and aptamers are rare, fluorophore labeling of Aptamers is essential for measurable signal generation, with signal intensity reflecting the binding affinity between Aptamers and targets (22). In 1996, Wang et al. (43) pioneered the first fluorescent aptasensor for small molecules by studying fluorophore-labeled RNA aptamers. Most labeled fluorescent aptasensors rely on FRET, where energy transfer occurs between a donor fluorophore and an acceptor quencher within a 1–10 nm distance, leading to fluorescence quenching. Target-induced conformational changes in Apts alter the donor-quencher proximity, terminating FRET and restoring fluorescence (44). However, conventional FRET systems are susceptible to pH and temperature fluctuations, which compromise fluorophore stability. To address this, nanomaterials with unique optical properties have been integrated to enhance robustness.

**Figure 2:**
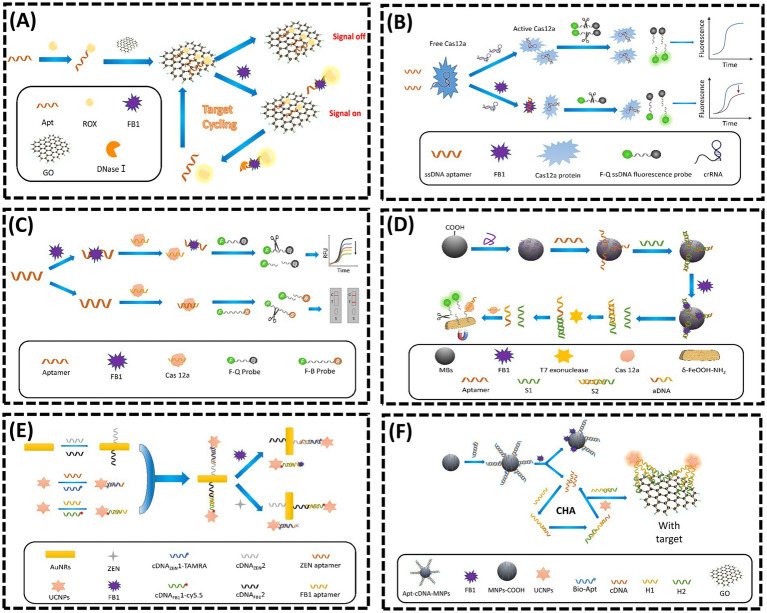
**(A)** Nuclease triggered “Signal-On” and amplified fluorescent sensing of FB1 incorporating GO and Aptamer. Adapted from (39), licensed under CC BY 4.0. **(B)** CRISPR-Cas12a-based aptasensor for FB1 detection. Adapted from (47) with permission from Elsevier. **(C)** A fluorescent aptasensor for detection for FB1 based on enzyme-assisted dual recycling amplification and 2D *δ*-FeOOH-NH_2_ nanosheets. Adapted from (49) with permission from Elsevier. **(D)** Dual-mode CRISPR/CAS12a-assisted fluorescent and lateral flow aptasensor for FBI detection. Adapted from (48) with permission from Elsevier. **(E)** Simultaneous determination of ZEN and FB1 based on gold nanorods and upconversion nanoparticle-based aptamers. Adapted from (53) with permission from Springer Nature. **(F)** A highly sensitive flurometric biosensor for FB1 detection based on UCNPs-GO and CHA. Adapted from (57) with permission from Elsevier.

Graphene oxide (GO), a two-dimensional nanomaterial with exceptional photoelectric properties, has been extensively utilized in FRET-based aptasensors for FB1 detection. In 2019, Wang et al. (45) designed a dual-target FRET sensor for AFB1 and FB1 using cross-linked CdTe quantum dots (QDs) as donors and magnetic GO/Fe_3_O_4_ nanocomposites as acceptors. The *π*–*π* stacking interaction between GO/Fe_3_O_4_ and QD-labeled Apts quenched fluorescence. Upon target binding, QDs were released from GO/Fe_3_O_4_, restoring fluorescence. Beyond quenching, GO also protects Apts from nuclease digestion. In 2022, Guo et al. (39) developed a novel nuclease-triggered “signal-on” fluorescent biosensor with signal amplification for FB1 detection using GO nanomaterials and a specific aptamer (Figure 2A). The aptamer was labeled with the fluorophore carboxy-X-rhodamine (ROX). When the ROX-modified aptamer was introduced into a GO solution, its fluorescence signal was significantly quenched due to the strong *π*–*π* stacking interaction between GO and the aptamer. In the presence of FB1, the aptamer preferentially bound to the target, forming a unique three-dimensional conformation (aptamer/FB1/ROX complex), which spatially separated the ROX fluorophore from the GO surface, thereby restoring fluorescence. Subsequently, nucleases digested the complex, releasing FB1 and causing the ssDNA and ROX to re-adsorb onto GO, resulting in fluorescence quenching. This sensor demonstrated excellent linearity for FB1 quantification within a dynamic range of 0.5–20 ng/mL, achieving a limit of detection (LOD) of 0.15 ng/mL. The design effectively combined target-induced conformational switching, GO-mediated fluorescence modulation, and enzymatic signal amplification to achieve sensitive and specific mycotoxin detection.

The Clustered Regularly Interspaced Short Palindromic Repeats (CRISPR) and CRISPR-associated protein (Cas) system, commonly referred to as CRISPR/Cas, has garnered significant attention in biosensing applications. In recent years, the trans-cleavage activity of the Cas12a/CRISPR RNA (crRNA) complex has emerged as a critical feature for detection strategies. Upon recognizing and binding to its target sequence, the CRISPR/Cas12a system exhibits nonspecific cleavage activity toward fluorophore-labeled complementary DNA (cDNA) probes, thereby restoring fluorescence signals (46). In 2023, Qiao et al. (47) proposed a dual-mode CRISPR/Cas12a-based aptasensor for rapid and sensitive detection of FB1 (Figure 2B). This design leveraged competitive binding between FB1 and crRNA for the FB1-specific aptamer F10. In the absence of FB1, F10 was bound to crRNA, triggering the trans-cleavage activity of Cas12a. This enzyme subsequently cleaved fluorophore-labeled probes, separating fluorophores from quenchers and generating an enhanced fluorescent signal. Conversely, when FB1 was existed, which was bound to F10, reducing the availability of ssDNA aptamers for crRNA binding. This process resulted in diminished Cas12a activation, fewer cleaved probes, and a significantly lower fluorescent signal. Similarly, Li et al. (48) employed the trans-cleavage activity of the Cas12a/crRNA complex to develop a fluorescent aptasensor for FB1, achieving a linear detection range of 10–1500 ng/mL and an LOD of 0.802 ng/mL (Figure 2C). In 2024, Li et al. (49) further advanced this field by integrating dual enzymatic cascade amplification with 2D *δ*-FeOOH-NH_2_ nanosheets for CRISPR/Cas12a-mediated FB1 detection (Figure 2D). In their system, FB1 was competed with a short DNA sequence (S1) for aptamer binding due to its high affinity. Higher FB1 concentrations released more S1, where it hybridized with a complementary strand (S2) to generate activator DNA (aDNA) via T7 exonuclease digestion. The released aDNA hybridized with crRNA, activating Cas12a’s cleavage activity. The activated Cas12a then cleaved ssDNA-FAM probes immobilized on *δ*-FeOOH-NH_2_ nanosheets, releasing fluorescent fragments and generating a quantitative signal. This approach combined enzymatic amplification with nanomaterial-based signal enhancement for improved sensitivity.

Lanthanide-doped upconversion nanoparticles (UCNPs), an emerging class of fluorescent nanomaterials, can convert low-energy near-infrared (NIR) light into high-energy visible emissions, offering unique advantages such as sharp emission peaks, high fluorescence purity, prolonged luminescence lifetimes, minimal background interference, robust chemical stability, and facile surface functionalization (50, 51). To date, several UCNP-based fluorescent sensors have been developed for monitoring FB1 levels in cereal-based foods. In 2013, Wu et al. (52) designed an aptasensor for FB1 detection by leveraging fluorescence resonance energy transfer (FRET) between NaYF_4_: Yb, Ho UCNPs and gold nanoparticles (AuNPs). In the absence of cDNA, the fluorescence of UCNPs was quenched via proximity to AuNPs. Upon FB1 introduction, the aptamer preferentially bound to FB1, displacing cDNA from magnetic nanoparticles (MNPs) and restoring fluorescence. In 2020, He et al. (53) developed a UCNP-based dual-target sensor for ZEN and FB1 detection (Figure 2E). Aptamers specific to ZEN and FB1 were hybridized with their corresponding cDNA strands to form double-stranded complexes, which were then conjugated to UCNPs. In the absence of targets, these UCNP-labeled complexes adsorbed onto gold nanorods (AuNRs), resulting in fluorescence quenching due to close proximity. However, the addition of ZEN or FB1 triggered aptamer-target binding, releasing the UCNP complexes from AuNRs and restoring fluorescence (54, 55). Despite these advantages, UCNPs suffer from self-quenching effects caused by surface defects, which compromise fluorescence intensity and detection sensitivity (56). To address this limitation, Qin et al. (57) introduced a novel biosensor in 2022 that integrated catalytic hairpin assembly (CHA), an enzyme-free and efficient amplification strategy, to enhance sensitivity for FB1 detection (58) (Figure 2F). In this system, FB1 binding to its aptamer triggered the release of cDNA strands, which were magnetically separated and employed as initiators for CHA amplification. This process generated H1-H2 double-stranded DNA (dsDNA) products, which were subsequently conjugated to UCNPs. The resulting UCNP-dsDNA complexes detached from GO surfaces, restoring fluorescence and effectively compensating for the sensitivity loss caused by UCNP self-quenching. This innovative approach demonstrated the synergistic integration of nucleic acid amplification techniques with UCNP-based signal transduction, highlighting a promising direction for ultrasensitive mycotoxin detection.

Recent advancements in fluorescent aptasensors have demonstrated remarkable progress through the integration of artificial intelligence (AI) and novel signal amplification strategies. A representative example is the recent research by Lin et al. (59), which developed a deep learning-assisted fluorescence single-particle aptasensor for ultrasensitive detection of FB1. This platform synergized entropy-driven catalysis (EDC) with *Thermus thermophilus* Argonaute (TtAgo)-mediated cleavage to achieve cascaded signal amplification. Upon FB1 binding, a trigger sequence was released from the aptamer, initiating the EDC cycle and generating abundant 5′-phosphorylated output strands. These strands were acted as guide DNA to activate TtAgo, which cleaved biotinylated signal probes conjugated to red fluorescence-encoded microspheres (RFEMs). After magnetic separation, the released RFEMs were imaged via confocal microscopy and quantified using a YOLOv9-based deep learning model. This approach achieved an LOD of 0.89 pg/mL with a linear range from 1 pg/mL to 100 ng/mL. Notably, the AI-driven image analysis eliminated manual counting errors and enhanced the detection throughput, processing images at 20 ms per frame with accuracy over 99%. Additionally, molecular docking-guided aptamer truncation optimized binding affinity, reducing synthesis costs while maintaining specificity. Through the integration of programmable nucleic acid amplification, nanomaterials, and AI, this study exhibited the great potential in improving the detection sensitivity and efficiency of fluorescence-based aptasensors.

#### 2.1.2 Colorimetric aptasensor

Colorimetric methods enable qualitative and semi-quantitative analysis through visually observable color changes, making them highly suitable for on-site rapid detection (44). Among colorimetric aptasensors, those leveraging the intrinsic optical properties of nanomaterials, particularly AuNPs, are most prevalent. These sensors exploit the size-dependent and distance-dependent optical characteristics of AuNPs, where colloidal state transitions induce distinct color shifts (60, 61). A dispersed AuNP solution appears red due to LSPR, while aggregation shifts the color to violet-blue as interparticle distances decrease and plasmonic coupling intensifies (62). Typically, aptamers adsorbed onto AuNP surfaces form protective layers that prevent salt-induced aggregation. Target binding induces aptamer conformational changes, causing desorption from AuNPs and subsequent aggregation, thereby triggering a red-to-blue color transition (61). Compared to other optical sensors, colorimetric systems offer advantages such as visual readout, low cost, operational simplicity, and compatibility with portable platforms, facilitating widespread practical applications. However, their dynamic range is often constrained by the detectable color variation, limiting precise quantitative analysis across broad concentrations. Additionally, environmental factors like temperature and pH may affect accuracy, necessitating strategies to enhance stability and signal amplification through material integration.

**Figure 3:**
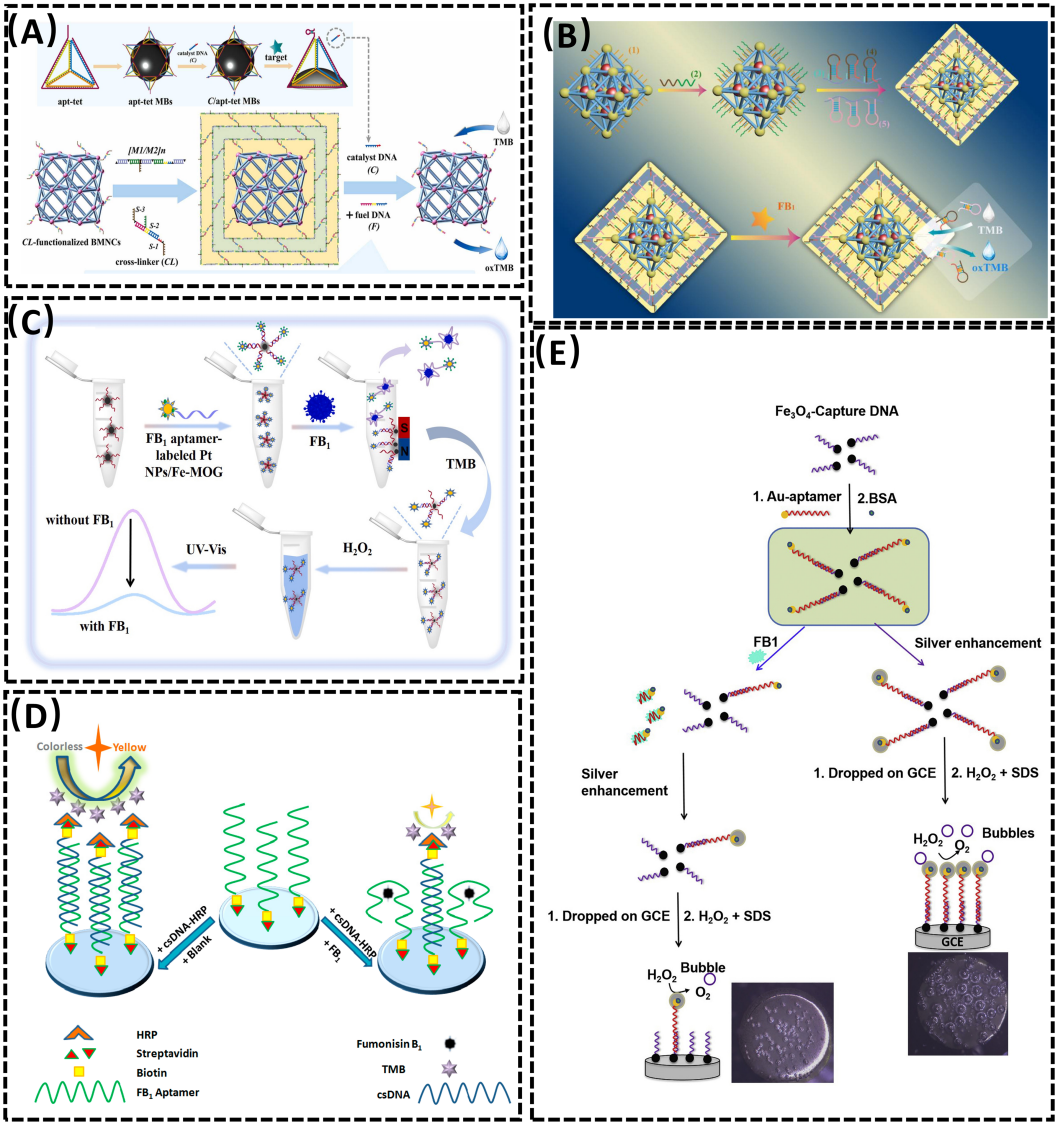
**(A)** Aptasensor for FBI detection based on the DNA tetrahedra-functionalized magnetic beads and DNA hydrogel-coated bimetallic MOFyzme. Reproduced from (40), with permission from Elsevier. **(B)** A colorimetric aptasensor based on the DNA hydrogel-coated MOF for FB1 determination. Reproduced from (64), with permission from Elsevier. **(C)** An aptasensor based on Pt nanoparticles-loaded on iron metal organic gel to FB1 analysis. Reproduced from (65), with permission from Elsevier. **(D)** Competitive HRP-linked colorimetric aptasensor for the detection of FB1. Reproduced from (68), licensed under CC BY 4.0. **(E)** Colorimetric aptasensor for FB1 detection by regulating the amount of bubbles in closed biopolar platform. Reproduced from (69), with permission from Elsevier.

Metal–organic frameworks (MOFs), characterized by porous structures, high surface areas, tunable pore architectures, and exceptional chemical/thermal stability, have emerged as promising substrates for sensor design (63, 64). In 2023, Sun et al. (40) developed a colorimetric aptasensor for FB1 detection by combining DNA tetrahedron-functionalized magnetic beads (MBs) with a DNA hydrogel-encapsulated Mn-Zr MOF nanozyme (MOFzyme) (Figure 3A). The DNA tetrahedron precisely controlled probe density and orientation. FB1 binding competitively displaced catalyst DNA from the aptamer, triggering hydrogel decomposition and exposing the MOFzyme. The released MOFzyme exhibited peroxidase-like activity, catalyzing chromogenic substrate oxidation to generate a colorimetric signal proportional to FB1 concentration. This system achieved a linear range of 5 × 10^−4^ to 50 ng/mL and an LOD of 0.38 pg/mL. In the same year, Sun et al. (64) engineered a “ship-in-a-bottle” porphyrin-embedded MOF (hemin@UiO-66-NH_2_) with enhanced peroxidase-mimetic activity and stability (Figure 3B). FB1 binding to the aptamer disrupted the hydrogel shell, releasing the MOF to catalyze substrate oxidation. The signal intensity correlated linearly with FB1 concentrations from 0.05 to 100 ng/mL, with an LOD of 0.024 ng/mL. Beyond MOFs, metal–organic gels (MOGs) have also been explored as sensor platforms. In 2022, Li et al. (65) designed a visual colorimetric aptasensor using an iron-based MOG (Fe-MOG) loaded with platinum nanoparticles (Pt NPs) as peroxidase mimics (Figure 3C). The Pt NPs/Fe-MOG composite was integrated with magnetic separation technology. Aptamer-conjugated carboxylated magnetic beads (cMBs) formed detection probes by binding to Pt NPs/Fe-MOG. FB1 presence triggered aptamer-target binding, reducing Pt NPs/Fe-MOG retention on cMBs and diminishing Tetramethylbenzidine (TMB) oxidation in the presence of H_2_O_2_, thereby attenuating the chromogenic signal. In contrast, absence of FB1 allowed full TMB coloration via unhindered Pt NPs/Fe-MOG activity. This approach combined nanomaterial-enhanced catalysis with magnetic separation for improved specificity and sensitivity in mycotoxin detection.

Horseradish peroxidase (HRP), a highly representative natural enzyme, has been widely employed as a catalytic component in biosensing systems due to its robust enzymatic activity and compatibility with signal amplification strategies (66, 67). In 2020, Tao et al. (68) developed a competitive HRP-linked colorimetric assay for FB1 detection by leveraging the biotin-streptavidin interaction (Figure 3D). Biotinylated FB1-specific aptamers were immobilized on streptavidin-coated microplates, while cDNA-HRP served as the sensing probe. In the presence of FB1, the toxin competitively displaced cDNA-HRP from the aptamer, resulting in minimal HRP retention and a light-yellow solution after substrate reaction. Conversely, in the absence of FB1, extensive cDNA-HRP binding to the aptamer yielded a dark yellow color due to amplified enzymatic catalysis. This inverse correlation between target concentration and colorimetric signal intensity enabled semi-quantitative FB1 analysis.

In the same year, Zheng et al. (69) reported a smartphone-integrated colorimetric biosensor based on a bubble-induced sensing mechanism (Figure 3E). The system utilized a bipolar electrode (BPE) fabricated by connecting a Pt wire (anode) in the reporting cell to a glassy carbon electrode (GCE, cathode) in the sample cell. FB1 detection relied on oxygen bubble formation modulated by target concentration. Upon FB1 binding, AuNP-Apt detached from Fe_3_O_4_ nanoparticle-complementary DNA (Fe_3_O_4_ NP-cDNA) surfaces, reducing silver deposition on the GCE while increasing Prussian blue (PB) deposition on the indium tin oxide (ITO) electrode. This differential deposition altered bubble generation and corresponding color patterns, which were quantified via smartphone imaging. This visual approach demonstrates promising potential for sensor development in terms of selectivity, reproducibility, and ease of fabrication, opening new avenues for portable mycotoxin detection.

#### 2.1.3 SERS aptasensor

**Figure 4:**
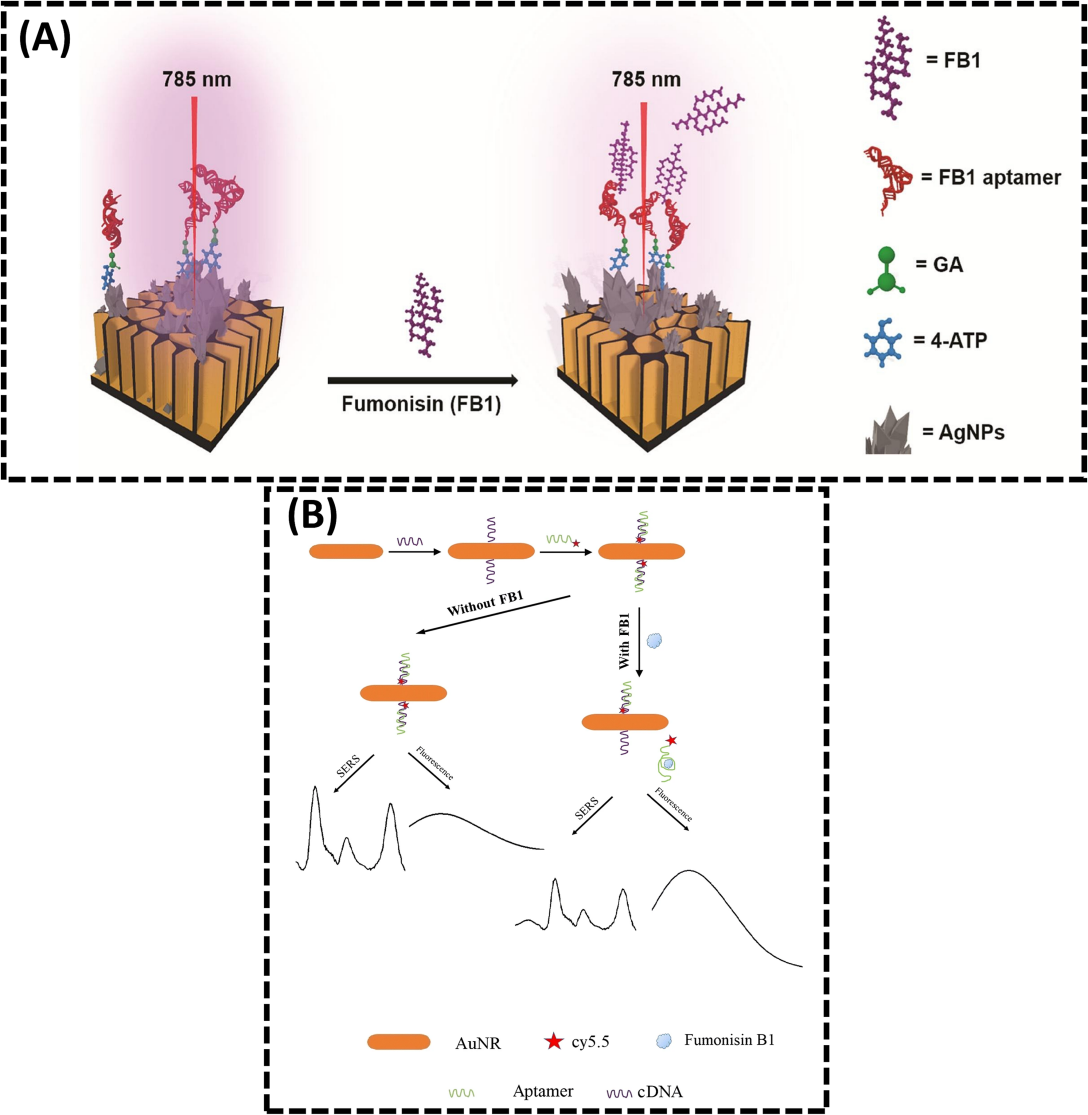
**(A)** Sensitive ratiometric detection of FB1 using an Ag-pSi SERS platform. Reproduced from (73), licensed under CC BY-NC-ND 4.0. **(B)** Aptamer and gold nanorod-based FB1 assay using both fluorometry and SERS. Reproduced from (42), with permission from Springer Nature.

The advancement of laser technology and nanomaterial fabrication has propelled the development of SERS, a powerful analytical technique capable of rapidly acquiring “fingerprint information” from target analytes. SERS enables facile identification and binding of aptamers through its exceptional sensitivity, superior resolution, and capacity for real-time monitoring of low-concentration targets (70). Studies have demonstrated that SERS significantly amplifies Raman signal intensity in dye/molecule/nanomaterial composites, making it a versatile tool for qualitative and quantitative detection of harmful residues in food matrices (71, 72). However, challenges such as weak SERS signals in complex samples and interference from coexisting compounds can compromise sensor selectivity and accuracy. Addressing these limitations requires the design of high-performance SERS substrates to enhance signal reproducibility and specificity. In 2025, Nirala et al. (73) engineered a ratiometric aptasensor using silver-coated porous silicon (Ag-pSi) as an optimized SERS substrate (Figure 4A). By tailoring surface porosity, pore morphology, and noble metal distribution, the Ag-pSi substrate achieved enhanced SERS effects. The substrate was functionalized with 4-aminothiophenol (4-ATP, a Raman reporter) and FB1-specific aptamers as biorecognition elements. Increasing FB1 concentrations progressively attenuated the optical response, with the sensor demonstrating high sensitivity (LOD = 0.05 ng/mL), a broad dynamic range (0.1–1000 ng/mL), and robust signal stability (RSD = 5.2%). Despite these advancements, the reliance on noble metal-based substrates raises cost concerns, limiting scalability (74).

To improve reliability, dual-signal detection strategies integrating complementary analytical modalities have emerged. He et al. (42) reported a dual-mode SERS-FL assay for FB1 quantification, leveraging inversely correlated signal responses (Figure 4B). In this system, FB1 binding induced aptamer dissociation from cDNA, simultaneously reducing SERS signals (due to nanoparticle aggregation) and enhancing fluorescence recovery (via probe displacement). This dual-signal approach cross-validates results, enhancing accuracy and reliability in complex sample analysis. Such hybrid methodologies highlight the potential of multimodal sensing platforms to overcome limitations inherent in single-mode detection systems.

### 2.2 EC aptasensor for FB1 detection

Electrochemical sensors are distinguished by their rapid response times, exceptional sensitivity, and high selectivity, making them widely applicable across diverse fields, particularly for detecting low-abundance small molecules such as mycotoxins. These sensors integrate the specific recognition capabilities of aptamers with electrochemical transduction, enabling the conversion of target analyte concentrations into measurable electrical signals for precise quantitative analysis (18). Photoelectrochemical (PEC) methods, EIS, and electrochemiluminescence (ECL), as advanced branches of EC technology, have garnered increasing attention for FB1 detection. PEC platforms leverage light-induced electron transfer processes to enhance signal amplification, while EIS monitors interfacial impedance changes induced by target binding. ECL, relying on light emission triggered by electrochemical reactions, offers ultra-sensitive detection with minimal background interference. These emerging techniques highlight the versatility and adaptability of electrochemical-based approaches in addressing the analytical challenges associated with mycotoxin monitoring (Table 1).

#### 2.2.1 Electrochemistry aptasensor

**Figure 5:**
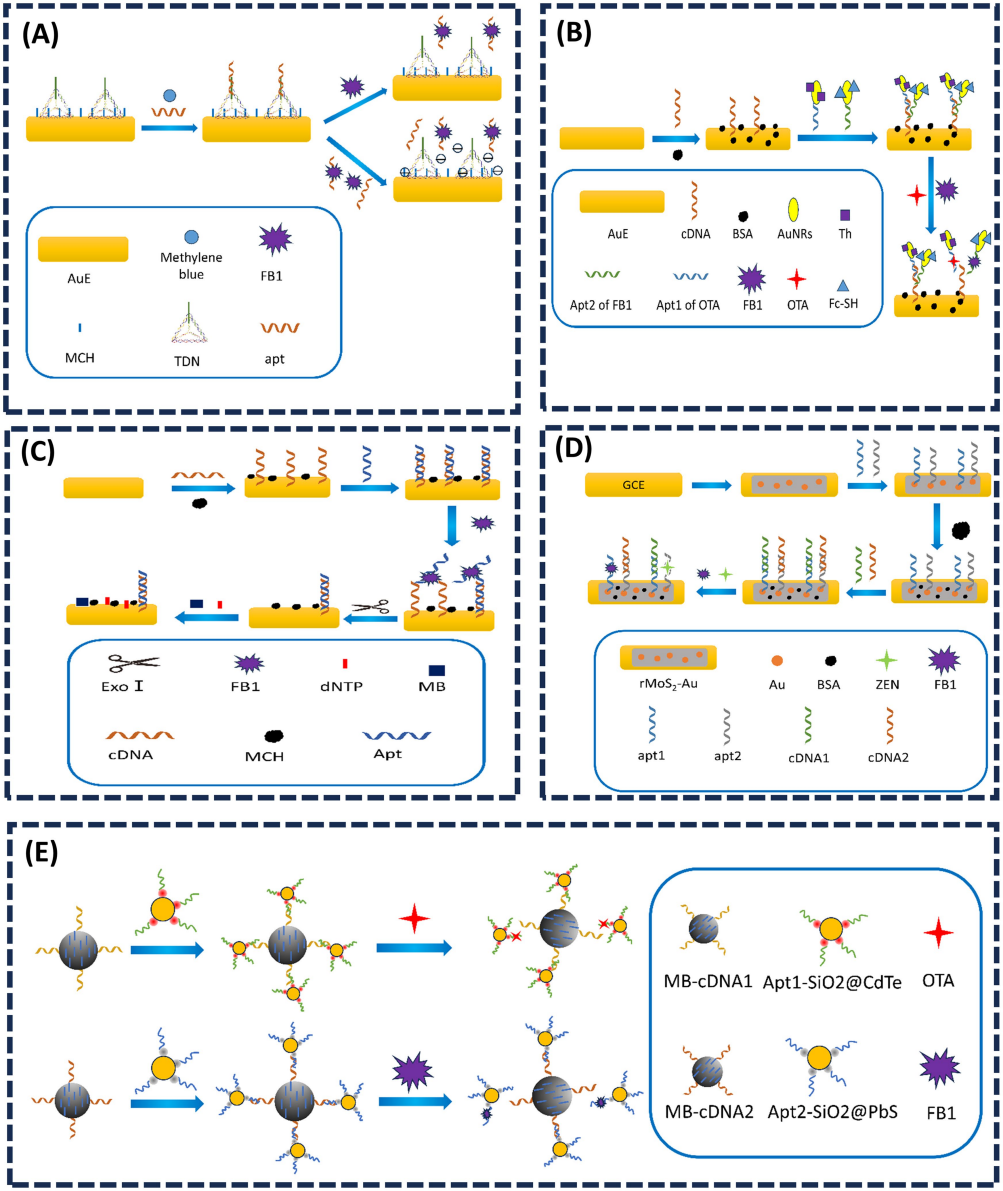
**(A)** Tetrahedral DNA nanostructure-enabled electrochemical aptasensor for detection of FB1. Adapted from (75), with permission from Elsevier. **(B)** Simultaneous electrochemical determination of OTA and FB1 with an aptasensor based on the use of a Y-shaped DNA structure on AuNRs. Adapted from (77), with permission from Springer Nature. **(C)** A novel electrochemical aptasensor for FB1 determination using DNA and exonuclease-I as signal amplification strategy. Adapted from (79), licensed under CC BY 4.0. **(D)** Dual-target electrochemical aptasensor based on co-reduced molybdenum disulfide and Au NPs (rMoS_2_-Au) for detection of ZEN and FB1. Adapted from (80), with permission from Elsevier. **(E)** Magneto-controlled aptasensor for simultaneous electrochemical detection of OTA and FB1 using metal sulfide quantum dots coated silica as labels. Adapted from (82), with permission from Elsevier.

The performance of recognition elements and substrate materials plays a pivotal role in determining the analytical capabilities of electrochemical sensors. Effective amplification of electrical signals is critical for enhancing sensitivity, while the structural morphology of the substrate directly influences sensor performance. In FB1 detection, overcrowding of multiple probes on the sensor interface can compromise sensitivity and stability. This challenge can be mitigated by introducing stable DNA nanostructures. Tetrahedral DNA nanostructures (TDNs), with their abundant binding sites and spatial hindrance, enable efficient signal amplification. In 2022, Dong et al. (75) reported a TDN-based electrochemical aptasensor for FB1 detection, where TDNs anchored on the electrode served as aptamer carriers, and methylene blue (MB) adsorption acted as the signal generator (Figure 5A). The sensor achieved a linear dynamic range of 0.5 fg/mL–1 ng/mL and an LOD of 0.306 fg/mL. For higher FB1 concentrations, auxiliary aptamers were introduced to reduce free FB1 levels, enabling extended linearity. Additionally, TDNs effectively suppressed nonspecific adsorption of free aptamers in complex samples. In 2023, Dong et al. (76) further advanced this approach by constructing a ratiometric aptasensor with MB anchored on TDNs and ferrocene (Fc) conjugated to Cdna. FB1 binding induced Apt-target hybridization, releasing TDNs and reducing spatial hindrance. This decreased MB signal (IMB) while increasing Fc signal (IFc), with the IFc/IMB ratio correlating linearly with FB1 concentrations from 0.1 to 100 pg/mL (LOD = 0.087 pg/mL). These studies demonstrate that TDNs enhance both sensitivity and dynamic range, offering a novel strategy for designing high-performance sensors with structured DNA architectures. Based on the concept of DNA nanostructures, Sun et al. (40) previously integrated DNA tetrahedrons with MOFs to improve stability in colorimetric sensors. Similarly, Wei et al. (77) designed a Y-shaped electrochemical aptasensor for simultaneous detection of OTA and FB1. AuNRs functionalized with thionine (Thi), SH-ferrocene (Fc), and SH-modified aptamers (SH-Apts) served as signal amplification and recognition elements (Figure 5B). cDNA immobilized on the Au electrode (AuE) surface hybridized with Apt1 and Apt2, forming a Y-shaped structure. In the absence of targets, abundant Apt1-AuNRs-Thi and Apt2-AuNRs-Fc generated dual redox currents. Target binding induced aptamer-toxin complex formation, releasing the probes and reducing currents. The sensor achieved linear ranges of 1.0 pg/mL – 100 ng/mL for both OTA and FB1, with LODs of 0.47 and 0.26 pg/mL, respectively.

Methylene blue (MB), a redox-active probe, interacts strongly with guanine-rich single-stranded or double-stranded DNA, making it ideal for electrochemical aptasensors (78). In 2019, Wei et al. (79) immobilized guanine-rich cDNA on an electrode, which hybridized with FB1-specific aptamers to form duplex DNA (Figure 5C). Without FB1, MB accumulated on the duplex, amplifying the electrochemical signal. Target binding displaced aptamers, and exonuclease-I (Exo-I) degraded single-stranded cDNA, reducing MB adsorption. The signal decrease correlated with FB1 concentrations from 1.0 × 10^−3^ to 1000 ng/mL (LOD = 0.15 pg/mL). Han et al. (80) designed reduced molybdenum disulfide gold nanoparticles (rMoS_2_-AuNPs), and Thi was employed to enhance the electrochemical signal. Accordingly, the proposed aptasensor possessed high sensitivity and selectivity for simultaneous detection of ZEN and FB1 (Figure 5D).

Quantum dots (QDs), a novel class of fluorescent nanomaterials, have garnered significant attention in toxin detection due to their superior optical properties, including broad absorption spectra, narrow and tunable emission bands, high fluorescence intensity, and exceptional photostability (19, 81). These attributes enable the development of sensors with amplified signal transduction mechanisms. In 2017, Wang et al. (82) designed a magnetic-controlled aptasensor for simultaneous electrochemical detection of OTA and FB1 in maize using CdTe or PbS QD-coated silica nanoparticles as labels (Figure 5E). Aptamers specific to OTA (Apt I) and FB1 (Apt II) were immobilized on magnetic beads (MBs). Upon target presence, Apt I and Apt II preferentially bound to OTA and FB1, displacing the QD-silica labels into the solution. The released labels reduced the electrochemical signal inversely proportional to toxin concentration. This sensor achieved broad detection ranges of 10 pg/mL–10 ng/mL for OTA and 50 pg/mL–50 ng/mL for FB1, demonstrating practical applicability in maize samples. Building on QD-based fluorescence resonance energy transfer (FRET), Wang et al. (45) previously utilized cross-linked CdTe QDs and GO quenching properties to develop a dual-target FRET sensor for AFB1 and FB1 detection.

#### 2.2.2 PEC aptasensor

**Figure 6:**
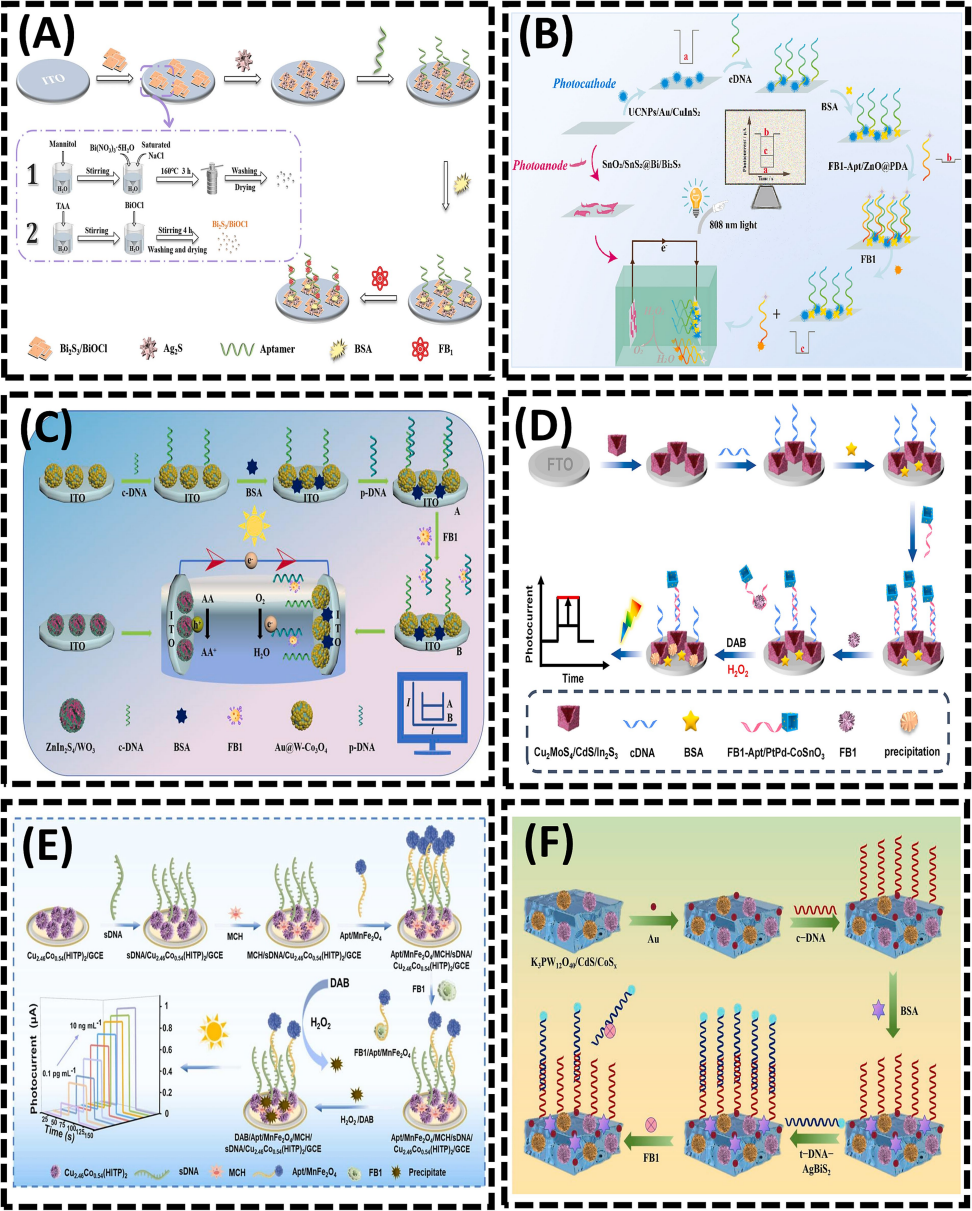
**(A)** Bi_2_S_3_/BiOCl heterojunction-based photoelectrochemical aptasensor for assay of fumonisinB1 via signal amplification with in situ grownAg_2_S quantum dots. Reproduced from (19), with permission from Springer Nature. **(B)** Near-infrared-driven dual photoelectrode photoelectrochemical sensing for FB1. Reproduced from (87), with permission from Elsevier. **(C)** Self-powered photoelectrochemical aptasensor for FB1 detection based on a Z-scheme ZnIn_2_S_4_/WO_3_ photoanode. Reproduced from (89), with permission from Elsevier. **(D)** Z-scheme Cu_2_MoS_4_/CdS/In_2_S_3_ nanocages heterojunctions-based PEC aptasensor for ultrasensitive assay of FB1 via signal amplification with hollow PtPd-CoSnO_3_ nanozyme. Reproduced from (90), with permission from Elsevier. **(E)** Ultrasensitive “signal-inversion” photoelectrochemical aptasensor based on semiconductive MOF integrated with the manganese ferrite nanozyme-regulation for detection of FB1. Reproduced from (91), with permission from Elsevier. **(F)** Quantitative analysis of FB1 using photoelectrochemical aptamer sensing strategy based on dual type II heterojunction K_3_PW_12_O_40_/CdS/CoS_x_. Reproduced from (92), with permission from Elsevier.

The detection mechanism of PEC sensors relies on variations in photocurrent and photopotential induced by physical and chemical interactions between targets and photochemical materials. High-efficiency photoactive materials are essential for achieving superior analytical performance in PEC sensors. Bismuth sulfide (Bi_2_S_3_), a significant photoactive material, exhibits remarkable environmental stability, exceptional charge transport properties, and a rapid photovoltaic response, making it a promising candidate for constructing PEC aptasensors (83). In 2024, Yu et al. (19) designed a PEC aptasensor using a layer-by-layer modification strategy (Figure 6A). By utilizing a bismuth sulfide/bismuth oxychloride (Bi_2_S_3_/BiOCl) composite, the combination of Bi_2_S_3_ and BiOCl not only expanded the light absorption range but also enhanced the intensity of the photocurrent response. *In situ*-grown silver sulfide (Ag_2_S) quantum dots further amplified the photocurrent response, significantly improving detection sensitivity. Upon illumination, electrons (e^−^) generated from the conduction band (CB) of Ag_2_S were directly transferred to the CB of Bi_2_S_3_, subsequently to the CB of BiOCl, and ultimately to an external detection circuit via ITO. This sensor achieved an LOD of 0.016 pg/mL for FB1. However, the rapid recombination of electron–hole pairs (e^−^/h^+^) in Bi_2_S_3_ limits its photocurrent intensity, hindering the attainment of ultra-high sensitivity (84). This challenge can be addressed through the construction of heterojunction (85, 86). In 2025, Guo et al. (87) substantially enhanced the light-harvesting capability of the photoanode by employing a SnO_2_/SnS_2_@Bi/Bi_2_S_3_ heterojunction (Figure 6B). The integration of such a photoanode into the photoelectric conversion sensing system markedly improved both photoelectric signals and sensing sensitivity. Owing to the strong binding affinity between FB1 and its aptamer (FB1-Apt), the presence of FB1 triggered the dissociation of FB1-Apt/ZnO@PDA from the photoelectrode, leading to photocurrent recovery. Bi_2_O_2_S has emerged as an excellent candidate for heterostructure construction due to its high photoelectric conversion efficiency. In 2025, Song et al. (88) developed a self-powered PEC aptasensor based on a Bi_2_O_2_S/Bi_2_S_3_ photoanode and Au@BiOI photocathode for FB1 detection (Figure 6C). Similarly, in 2024, Ren et al. (89) proposed a self-powered PEC aptasensor using Au@W-Co_3_O_4_ as a photocathode to provide the sensing interface and ZnIn_2_S_4_/WO_3_ as a photoanode to amplify cathodic signals, achieving highly sensitive FB1 detection. Under visible light irradiation, the ZnIn_2_S_4_/WO_3_ photoanode enhanced electron transfer rates, thereby contributing to signal amplification at the photocathode. Meanwhile, the Au@W- Co_3_O_4_ photocathode served as the sensing interface, effectively reducing the probability of false positives.

In 2023, Wei et al. (90) constructed a PEC sensing platform for FB1 detection using Cu_2_MoS_4_/CdS/In_2_S_3_ as the photosensitive substrate combined with PtPd alloy-decorated hollow CoSnO_3_ nanoboxes (denoted as PtPd-CoSnO_3_) nanozymes (Figure 6D). In 2024, Li et al. (91) integrated a competitive biosensing strategy with enzyme-assisted biocatalytic precipitation reaction (EABPR) technology to design a novel “signal-reversal” PEC aptasensor (Figure 6E). Upon FB1 detection, the binding of FB1 to its aptamer triggered the formation of a G-quadruplex through their specific recognition, leading to the release of the FB1/Apt/MnFe_2_O_4_ complex from the electrode surface. In the presence of H_2_O_2_, MnFe_2_O_4_ catalyzed the oxidation of DAB (3,3′-diaminobenzidine) to form insoluble precipitates. Consequently, as the FB1 concentration increased, the amount of Apt/MnFe_2_O_4_ retained on the electrode decreased, resulting in reduced precipitate deposition. This diminished the inhibition of electron transfer at the electrode/electrolyte interface, thereby amplifying the photocurrent signal. In 2025, Wang et al. (92) developed a PEC aptasensor using K_3_PW_12_O_40_/CdS/CoSₓ as the substrate, where the aptamer (t-DNA) labeled with AgBiS_2_ served as a signal amplifier for sensitive FB1 detection (Figure 6F). The formation of a K_3_PW_12_O_40_/CdS/CoSₓ dual heterojunction facilitated enhanced visible light absorption and accelerated electron transfer kinetics. The introduction of t-DNA-AgBiS_2_ induced a measurable change in photocurrent intensity. Upon FB1 addition, the specific recognition interaction between FB1 and t-DNA triggered the detachment of t-DNA-AgBiS_2_ from the electrode surface. This process “turned on” the photocurrent signal, enabling FB1 quantification through photocurrent analysis.

#### 2.2.3 EIS

As an intuitive electrochemical technique, EIS characterizes changes in the electron transfer resistance (R_et_) at the electrode interface. Due to its simplicity, rapidity, cost-effectiveness, high sensitivity, and potential for miniaturization, EIS-based aptasensors have gained increasing prominence in the on-site monitoring of mycotoxins (93).

**Figure 7:**
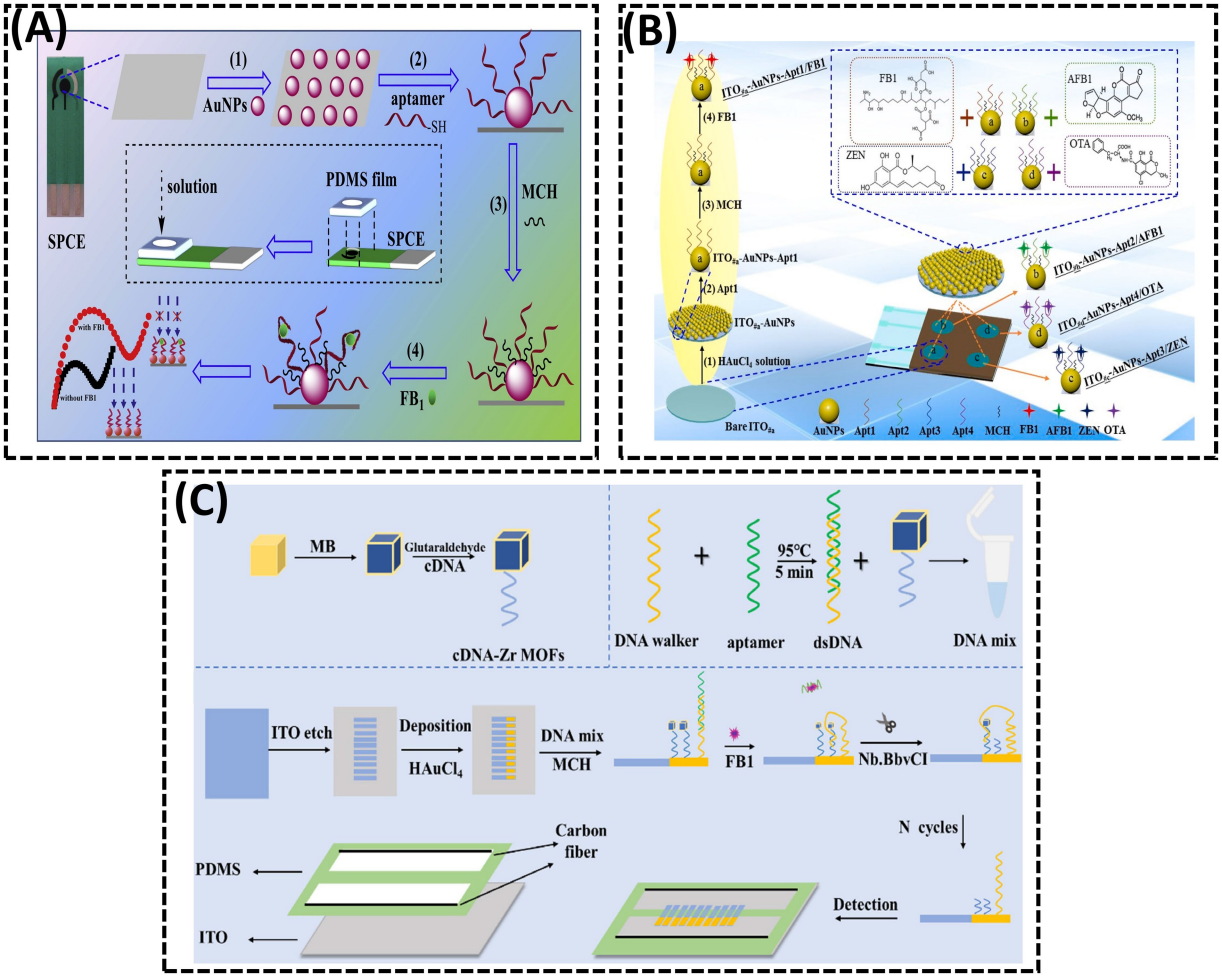
**(A)** A disposable aptasensing device for label-free detection of FB1 by integrating PDMS film-based micro-cell and screen-printed carbon electrode. Reproduced from (97), with permission from Elsevier. **(B)** Fabrication of a disposable aptasensing chip for simultaneous label-free detection of FB1, AFB1, ZEN, and OTA. Reproduced from (17), with permission from Elsevier. **(C)** Visual measurement of FB1 with bipolar electrodes array-based electrochemiluminescence biosensor. Reproduced from (101), licensed under CC BY 4.0.

AuNPs are widely employed in the fabrication of EIS biosensors owing to their exceptional properties (94, 95). In 2015, Chen et al. (93) utilized electrochemically deposited AuNPs to enhance the immobilization density of DNA probes (96). A thiolated aptamer specific to FB1 was anchored onto AuNPs coated on the GCE. During the incubation with FB1, the target was captured on the GCE surface via specific recognition between FB1 and its aptamer. As the concentration of FB1 increased, more targets bound to the electrode, significantly inhibiting electron transfer between the electrolyte and the electrode, thereby elevating the interfacial resistance. In 2017, Ren et al. (97) electrodeposited dense AuNPs onto the working electrode of a screen-printed carbon electrode (SPCE) to immobilize the FB1 Apt, followed by blocking excess active sites on AuNPs with 6-mercapto-1-hexanol (MCH) (Figure 7A). In the absence of FB1, the Apt allowed the redox probe [Fe(CN)_6_]^4−/3−^ to access the electrode surface (98). However, the presence of FB1 triggered the formation of an FB1-Apt complex, inducing a conformational transition of the aptamer into a G-quadruplex structure (99). This structural change increased the charge transfer resistance (Ret) at the interface. In 2023, Qian et al. (17) enhanced the sensitivity of EIS-based aptasensors by electrodepositing AuNPs onto the working electrode and self-assembling thiol-modified Apt via Au-S bonds, enabling the simultaneous detection of four mycotoxins (Figure 7B). When the four mycotoxins (FB1, AFB1, ZEN, and OTA) coexisted, distinct EIS signal variations facilitated label-free multiplex detection in a single sample. The LODs for FB1, AFB1, ZEN, and OTA were 2.47, 3.19, 5.38, and 4.87 ng/mL, respectively. This integrated approach eliminated the need for modifying multiple probes on a single sensing interface, thereby creating an interference-free detection platform.

#### 2.2.4 Electrochemiluminescence (ECL)

ECL, a sensitive optical analytical technique that integrates electrochemical and luminescent properties, has attracted considerable attention due to its high sensitivity, spatial controllability, and unique advantages in bioanalysis. In 2014, Zhao et al. (100) developed an AuNP-driven ECL aptasensor that incorporated ionic iridium (Ir) complexes for the sensitive detection of FB1 (Figure 7C). In 2023, Jin et al. (101) reported a BPE-based arrayed ECL platform for FB1 detection. A DNA hybrid solution containing aptamer-DNA walker duplexes and methylene blue (MB)-labeled cDNA, immobilized on Zr-based metal–organic frameworks (MB@Zr-MOFs), was anchored to AuNPs on the BPE cathode. Due to the accumulation of abundant MB molecules on the BPE cathode, the MB@Zr-MOFs induced a significant enhancement of the ECL signal. In the presence of FB1, the DNA walker was activated, leading to the continuous release of MB from the electrode surface with the assistance of a nicking endonuclease. This process generated a pronounced quenching effect on the ECL signal. A linear relationship was observed between the ECL signal intensity and the logarithm of FB1 concentration within the range of 5 × 10^−5^ to 0.5 ng/mL.

## 3 Challenges and perspectives

Although aptamer-based sensors have demonstrated excellent performance in detecting FB1 contamination, several challenges remain to be addressed:

1. Limitations of multiplex detection. Although significant progress has been made in the individual analysis of FB1, research on the simultaneous detection of multiple mycotoxins remains considerably less advanced. This gap primarily arises from the challenges associated with developing recognition elements that exhibit specificity for various mycotoxins and designing effective multiplex detection strategies (74).
2. Interference from fluorescent labeling. In fluorescence detection, naturally occurring fluorescent biomolecules or aptamers are rare, necessitating the use of fluorophore-modified aptamers. However, the presence of fluorophores and quenchers can alter the binding affinity between aptamers and their targets. Therefore, mitigating or eliminating the impact of these modifications on aptamer affinity is a critical research direction for fluorescence-based detection technologies.
3. Sample complexity and signal reliability. Real-world samples often contain complex matrices that can interfere with sensor performance. Ratiometric and dual-mode sensing strategies have the potential to enhance detection accuracy. However, to date, only one study has reported a dual-signal sensor (combining SERS and FL signals) for FB1 detection (42). Furthermore, the development of ratiometric sensors remains insufficient. Advancing dual-signal sensing platforms is essential for improving the efficiency and reliability of FB1 analysis.
4. Environmental sensitivity of colorimetric sensors. Colorimetric aptasensors are highly sensitive to environmental factors such as temperature, pH, and ionic strength, which can compromise their sensitivity (102). Developing robust strategies to minimize environmental interference would significantly enhance the detection of FB1 and other mycotoxins.
5. Integration of nanomaterials. Advanced nanomaterials, including GO (39), MOF (40), UCNP (53), QD (82), and AuNP (93), have been integrated into aptasensors to amplify signals, enhance signal transduction, and provide abundant aptamer-binding sites. Further exploration of novel nanomaterials is essential to optimize sensor performance.
6. Portability and Automation: The increasing demand for rapid, on-site detection of FB1 necessitates the development of miniaturized, cost-effective, and user-friendly aptasensors (103). Integrating AI into sensor systems could revolutionize data processing, particularly for high-throughput sample analysis. Portable platforms, such as smartphone-coupled sensors, represent a promising frontier for future innovations in aptasensor technology.

## 4 Conclusion

Fumonisins are mycotoxins commonly found in maize, wheat, oats, and related products. FB1, the most prevalent and toxic variant, causes significant food contamination and poses serious risks to public health. Therefore, monitoring FB1 levels in food is critical. Traditional analytical methods, such as LC–MS, HPLC-FLD, and ELISA, achieve high sensitivity but have limitations, including reliance on expensive instrumentation, complex sample pretreatment, and labor-intensive procedures. To address these challenges, nucleic acid aptamer-based sensors have emerged as a promising alternative for FB1 detection. Aptamers, which serve as efficient biorecognition elements, are central to the construction of these sensors. Over three decades of advancements in the SELEX technique, particularly through integration with novel technologies, have significantly enhanced the stability, affinity, and specificity of aptamers. Commercially available aptasensors primarily employ fluorescence, colorimetry, or voltammetry, each with distinct advantages and limitations. For instance, colorimetric aptasensors, which allow for visual or semi-quantitative analysis through naked-eye detection, are ideal for on-site screening. In contrast, fluorescent-based and electrochemical-based aptasensors are recommended for scenarios that require ultra-high sensitivity. The selection of the optimal platform should align with specific detection requirements, balancing analytical performance, cost, and operational simplicity.

## Glossary

FB1: Fumonisin B1
ELISA: Enzyme-linked immunosorbent assay
AFB1: Aflatoxin B1
OTA: Ochratoxin A
ZEN: Zearalenone
BRE: Bio-recognition element
IC-ELISA: Indirect competitive enzyme-linked immunosorbent assay
MIPSPE: Molecularly imprinted polymer solid-phase extraction
FL: Fluorescence
SPR: Surface plasmon resonance
Apt: Aptamer
ROX: Carboxy-X-rhodamine
MBs: Magnetic beads
UCNPs: Upconversion nanoparticles
NIR: Near-infrared
CHA: Catalytic hairpin assembly
MOFzyme: Metal–organic frameworks-based nanozyme
MOG: Metal organic gel
BPE: Bipolar electrodes
PB: Prussian Blue
TDN: Tetrahedral DNA nanostructures
FC: Ferrocene
ECL: Electrochemiluminescence
BiOCl: Bismuth oxychloride
R_et_: Electron transfer resistance
ITO: Indium Tin Oxide
EABPR: Enzyme-assisted biocatalytic precipitation reaction
EABPR: Enzyme-assisted biocatalytic precipitation reaction
STE: Signal transduction element
HPLC: High performance liquid chromatography
LC–MS: Liquid chromatography-mass spectrometry
TLC: Thin layer chromatography
GC–MS: Gas chromatography-mass spectrometry
SELEX: Systematic Evolution of Ligands by Exponential Enrichment
SERS: Surface-enhanced Raman spectroscopy
FRET: Fluorescence resonance energy transfer
GO: Graphene oxide
QDs: Quantum dots
CRISPR: Clustered regularly interspaced short palindromic repeat
LOD: Limit of detection
aDNA: Activator DNA
AuNPs: Gold nanoparticles
MNP: Magnetic nanoparticles
dsDNA: Double-stranded DNA
Pt NPs: Platinum nanoparticles
TMB: Tetramethylbenzidine
HRP: Horseradish peroxidase
MB: Methylene blue
Thi: Thionine
EIS: Electrochemical impedance spectroscopy
GCE: Glass carbon electrode
SPCE: Screen-printed carbon electrode
CB: Conduction band
e−/h+: electron/hole pair
RSD: Relative standard deviation
